# AMPed up immunity: 418 whole genomes reveal intraspecific diversity of koala antimicrobial peptides

**DOI:** 10.1007/s00251-024-01368-2

**Published:** 2025-01-08

**Authors:** Cleopatra Petrohilos, Emma Peel, Luke W. Silver, Katherine Belov, Carolyn J. Hogg

**Affiliations:** 1 School of Life and Environmental Sciences, The University of Sydney, Sydney, NSW Australia; 2 Australian Research Council Centre of Excellence for Innovations in Peptide & Protein Science, The University of Sydney, Sydney, NSW Australia

**Keywords:** Cathelicidins, Defensins, Immune genes, Conservation genomics

## Abstract

Characterising functional diversity is a vital element to understanding a species’ immune function, yet many immunogenetic studies in non-model organisms tend to focus on only one or two gene families such as the major histocompatibility complex (MHC) or toll-like receptors (TLR). Another interesting component of the eukaryotic innate immune system is the antimicrobial peptides (AMPs). The two major groups of mammalian AMPs are cathelicidins and defensins, with the former having undergone species-specific expansions in marsupials. Here, we utilised data from 418 koala whole genomes to undertake the first comprehensive analysis of AMP diversity across a mammalian wildlife species’ range. Overall, allelic diversity was lower than other immune gene families such as MHC, suggesting that AMPs are more conserved, although balancing selection was observed in PhciDEFB12. Some non-synonymous SNPs in the active peptide are predicted to change AMP function through stop gains, change in structure, and increase in peptide charge. Copy number variants (CNVs) were observed in two defensins and one cathelicidin. Interestingly, the most common CNV was the duplication of PhciCATH5, a cathelicidin with activity against chlamydia, which was more common in the southern part of the species range than the north. AMP copy number is correlated with expression levels, so we hypothesise that there is a selective pressure from chlamydia for duplications in PhciCATH5. Future studies should use phenotypic metadata to assess the functional impacts of this gene duplication.

**Supplementary Information:**

The online version contains supplementary material available at 10.1007/s00251-024-01368-2.

## Introduction

Immune genes are some of the fastest evolving in the genome due to the co-evolutionary arms race between hosts and pathogens (Barreiro & Quintana-Murci 2010; Trowsdale & Parham 2004). Although balancing selection frequently results in many immune genes being highly polymorphic, this is not uniform across all immune gene families (Mukherjee et al. 2009; Vinkler et al. 2023). Characterising functional diversity is an important component of understanding species’ biology and is particularly important in threatened species management where immunogenetic variation has been shown to influence disease susceptibility in many species (Elbers et al. 2018; Morrison et al. 2020). However, much of the research in non-model organisms to date has only focused on a small number of immune gene families such as the major histocompatibility complex (MHC) and toll-like receptors (TLR) (Bagheri & Zahmatkesh 2018; Brouwer et al. 2010; Grueber et al. 2014; Minias et al. 2019; Minias & Vinkler 2022; Vinkler et al. 2023).

More recently, other immune genes such as antimicrobial peptides (AMPs) have been investigated (Chapman et al. 2016; Hellgren 2015; Schmitt et al. 2017). AMPs are small, cationic molecules with broad spectrum antimicrobial activity. They are an ancient component of the eukaryotic immune system and are expressed by both plants and animals (Zasloff 2002). The two major families of mammalian AMPs are cathelicidins and defensins (Gallo et al. 2002). Both are encoded as precursor molecules composed of three domains: a signal sequence, a propeptide sequence, and an antimicrobial domain. Upon activation, the antimicrobial domain is cleaved off to create the active mature peptide (Selsted & Ouellette 2005; Zanetti 2005).

AMPs generally exhibit lower overall diversity than other immune genes, with only one or two haplotypes being common across a population (Chapman et al. 2016; Gilroy et al. 2016; Hellgren 2015; Schmitt et al. 2017). Immune genes such as MHC benefit from high levels of polymorphism as this enables them to bind to a wide variety of antigenic peptides (Spurgin & Richardson 2010). In contrast, AMPs are characterised by their broad-spectrum activity against a diverse range of microbes. This is largely mediated by their physicochemical properties such as net cationic charge and the ability to adopt an amphipathic structure (Zasloff 2002). These properties enable the peptides to electrostatically bind to negatively charged bacterial membranes and disrupt their integrity, leading to cell death (Zasloff 2002). However, they also likely constrain nucleotide polymorphisms given physiochemical properties are tightly linked to function.

Characterising diversity in AMPs is important as even minimal amino acid changes can drastically alter the physicochemical and antimicrobial properties of peptides (Hellgren et al. 2010; Higgs et al. 2007; Meade et al. 2008). To date, intraspecific AMP diversity has only been investigated in birds and agricultural animals (Brahma et al. 2015; Chapman et al. 2016; Gillenwaters et al. 2009; Gilroy et al. 2016; Hellgren 2015; Ishige et al. 2021; Monteleone et al. 2011; Schmitt et al. 2017). Cathelicidin intraspecific diversity has not yet been investigated in wildlife, with most studies instead exploring defensin diversity in one or two populations (with a total sample size ranging from 5 to 160) (Gilroy et al. 2016; Hellgren 2015; Schmitt et al. 2017). The most comprehensive study has involved mallards (*Anas platyrhynchos*) from global populations (*n* = 274 from a single location in Sweden and *n* = 190 from 16 other geographic locations), although only five genes were included in this analysis (Chapman et al. 2016). Collectively, these studies suggest that most defensins are either monomorphic or primarily consist of one major allele that is predominant across a population.

However, some diversity has been observed within AMPs, at both nucleotide and copy number levels (Supplementary File 1). For example, the mallard defensin AvBD10 consists of 41 alleles. Only two alleles are present at high frequencies in most populations but their frequencies can vary between geographic regions (Chapman et al. 2016). The domestic water buffalo (*Bubalus bubalis*) expresses seven cathelicidins with one (CATHL4) having high polymorphism at both the nucleotide and copy number level (Brahma et al. 2015). As this study only involved 25 individual animals from a single slaughterhouse, it is unknown whether this differs between populations. Defensins also exhibit high variation in copy number between individuals in humans (Hollox et al. 2003; Linzmeier & Ganz 2005), with variation shown to influence disease susceptibility (Hollox 2009; Hollox et al. 2008).

All these studies have relied on species-specific primers to amplify genes of interest. This means they have been restricted to studying a small number of genes (typically four to six) that represent only a fraction of a species’ AMP repertoire. As whole genome sequencing (WGS) costs have decreased, our ability to investigate functional diversity in non-model organisms across multiple gene families has increased (Fuentes‐Pardo & Ruzzante, 2017). WGS has enabled exploration of the entire range of AMP genes in a species’ genome rather than being limited to a small number of genes using more conventional analytical methods.

Marsupials are a particularly interesting model species as they encode a diverse repertoire of AMPs. The Tasmanian devil (*Sarcophilus harrisii*) has seven cathelicidins (Peel et al. 2016), gray short-tailed opossum (*Monodelphis domestica*) has 19 (Cho et al. 2020), and the koala (*Phascolarctos cinereus*) has ten (Peel et al. 2021), whilst humans and mice only have a single cathelicidin gene (Ramanathan et al. 2002). This high diversity in marsupials is often attributed to their unique reproductive biology (Peel et al. 2017), characterised by a short gestation period and birthing of highly altricial young that are immunologically naive (Old & Deane 2000). Marsupial neonates are often exposed to a variety of pathogens as they complete their development in the non-sterile pouch environment (Maidment et al. 2023; Weiss et al. 2021). During this period, they depend on passive immunity conferred by the mother, including AMPs delivered through pouch secretions, maternal licking, and milk (Edwards et al. 2012). The selective pressure of this environment may have encouraged the species-specific expansion observed in the cathelicidins of marsupials.

Many marsupial AMPs exhibit antimicrobial activity in vitro against a range of bacteria, fungi, and viruses, including multidrug-resistant strains (Cho et al. 2020; Peel et al. 2016, 2017; Wang et al. 2011). Interestingly, marsupial cathelicidins are also active against pathogens of conservation concern. For example, the koala cathelicidin PhciCATH5 is active against the bacterium *Chlamydia pecorum* (Peel et al. 2021), the main causative agent of chlamydiosis and a major threatening process for the species (Polkinghorne et al. 2013). Chlamydiosis is an endemic disease with devastating effects on koala populations that can lead to blindness and infertility (Polkinghorne et al. 2013). Similarly, Tasmanian devil cathelicidins exhibit anticancer activity against devil facial tumour disease (DFTD), a contagious cancer that has decimated populations throughout the species’ range (Petrohilos et al. 2023). These findings make marsupial AMPs promising candidates for drug discovery and development against a range of pathogens of concern, for both humans and wildlife.

In 2021, the Koala Genome Survey sequenced 430 koala genomes across the east coast of Australia to improve understanding of the species’ genomic diversity (Hogg et al. 2023). Here, we used 418 individuals from this unique dataset to conduct the first comprehensive analysis of AMP diversity across a wildlife mammal species’ range, ~ 700 000 km^2^ in the instance of koalas. Our aims were to (i) characterise baseline level of nucleotide and copy number diversity amongst koala AMPs across their geographic range and (ii) use *in silico* methods to predict functional effects of SNPs and identify targets for future research.

## Methods

We downloaded 418 aligned koala BAM files from the Amazon Web Services (AWS) Open Data Program (https://awgg-lab.github.io/australasiangenomes/species/Phascolarctos_cinereus.html). Captive koalas (*n* = 12) were excluded from our dataset as they were deemed not to represent natural genetic diversity. A multi sample VCF file was created using the Dragen gVCF genotyper in the non-iterative mode on a Dragen V4 server. The Dragen Joint Genotyping Pipeline (v3.9.5) was used to run joint genotyping on Illumina’s Basespace portal.

Published koala defensin (Jones et al. 2017) and cathelicidin (Peel et al. 2021) sequences were annotated in the current genome assembly (GCA_002099425.1_phaCin_unsw_v4.1) (Johnson et al. 2018) using BLAST v2.2.30 (Altschul et al. 1990). Exons were extracted using bedtools (version 2.29.2) and manually checked to ensure consensus with published sequences (Jones et al. 2017; Peel et al. 2021). In total, 38 AMPs were used in this study: seven cathelicidins, two alpha defensins (including one partial sequence), and 29 beta defensins (including 21 partial sequences). Although it is possible these partial sequences represent pseudogenes, we included them as very short sequences that are difficult to identify using homology-based algorithms. The first exon of marsupial defensins has an average length of 69 bp, and so many marsupial defensins have only been annotated as partial sequences (Peel et al. 2024). As the second exon contains the active peptide sequence that is of most interest, many studies have only focused on this region when characterising diversity (Chapman et al. 2016). The PhciDEFB10 sequence annotated in this study differs to the published sequence (Jones et al. 2017), which we attribute to the different versions of the genome assembly. The coordinates and sequences of the AMPs used in this study are listed in Supplementary File 2.

The whole genome joint genotyped multi sample VCF file was filtered to only include biallelic variants within the AMP genes using gatk (version 4.2.0.0) SelectVariants. Gatk (version 4.2.0.0) VariantFiltration was then used to exclude variants with QUAL < 40, MQ < 40.00, MQRankSum >|12.5|, FS > 60.00, QD < 1.5, ReadPosRankSum >|8.00|. AMPs that did not contain any SNPs were excluded from further analysis. Variants were annotated using Annovar (version 20180416) and classified as intronic or exonic, with exonic variants categorized as synonymous or non-synonymous.

Alleles were determined by first using gatk (version 4.2.1.0) FastaAlternateReferenceMaker to obtain the nucleotide sequence for all AMPs in each individual. Seqphase (Flot 2010) was used to convert sequences into PHASE format; PHASE (version 2.1.1) was then used to determine alleles and estimate allele frequencies for each gene. Seqphase was then used to convert the output into fasta format. Alleles that occurred in less than two individuals were excluded as potential errors. Alleles for each gene are saved in Supplementary File 3. Sequences with unresolved alleles were removed, and Tajima’s *D* was calculated using DnaSP 6 (Rozas et al. 2017).

Alleles that contained non-synonymous SNPs in the active peptide region (exon four of cathelicidins and exon two of defensins) were tested *in silico* to predict the functional effect. The webserver https://protcalc.sourceforge.net/ was used to calculate change in peptide charge; the CSM-peptides webserver (Rodrigues et al. 2022) was used to predict antibacterial, antiviral, anticancer, and anti-inflammatory activity; and the AntiFungal webserver (Zhang et al. 2021) was used to predict antifungal activity. Three-dimensional structures of the active peptides with non-synonymous SNPs were predicted using RoseTTAFold (Baek et al. 2021) and visualized using Chimera 1.18 (Pettersen et al. 2004) (Supplementary File 7 and Supplementary File 8).

Datamonkey webserver (Delport et al. 2010; Pond & Frost 2005; Weaver et al. 2018) was used to test for residues under positive or negative selection using three tests: fixed effects likelihood (FEL) (Kosakovsky Pond & Frost 2005), mixed effects model evolution (MEME) (Murrell et al. 2012), and A Fast, Unconstrained Bayesian AppRoximation for Inferring Selection (FUBAR) (Murrell et al. 2013). As Datamonkey requires a minimum of three sequences, only AMPs with three or more alleles were tested for selection. Only the coding sequence was used for testing, and sites were considered under selection if they were supported by two or more of the tests.

**Fig. 1:**
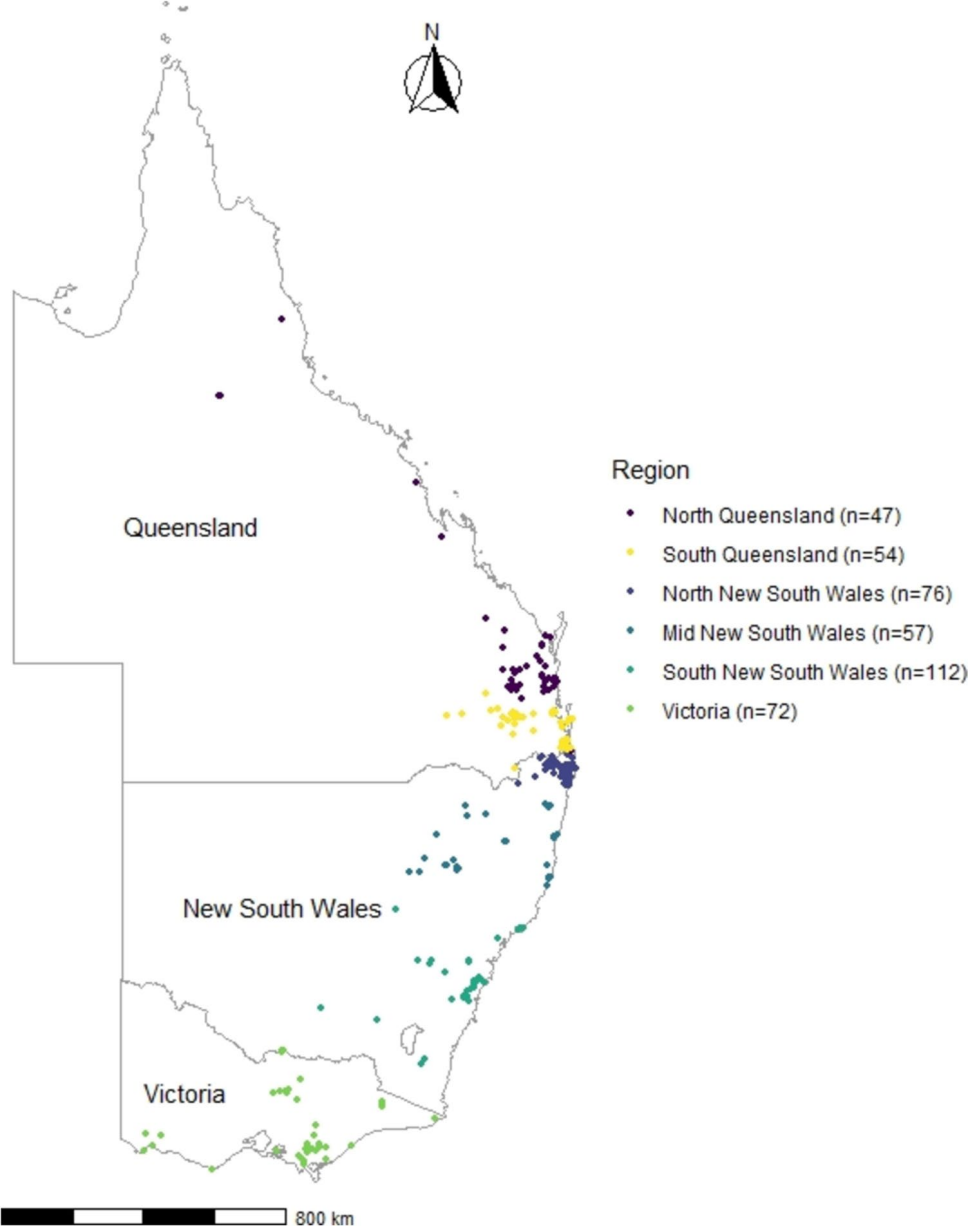
Sampling locations coloured by geographic subregions

CNVnator (version 0.4.1) was used to call copy number variants (CNVs) on the BAM files using a bin size of 1000 bp. Low-quality calls were removed, including those with q0 > 0.5 (indicating that more than 50% of the reads supporting the CNV had zero mapping quality), *e*-value > 0.05, and size < 1 kb or > 5 Mb. Any CNVs that overlapped with regions containing AMPs were collated into a single file (Supplementary File 4). Samples were then divided into six geographic regions to determine the distribution of CNVs: North Queensland (QLD) (samples collected north of Brisbane River) (*n* = 47), South QLD (samples collected south of Brisbane River) (*n* = 54), North New South Wales (NSW) (samples collected north of Clarence River) (*n* = 76), Mid NSW (samples collected south of Clarence River and north of Hunter Valley (*n* = 57), South NSW (samples collected south of Hunter Valley) (*n* = 113), and Victoria (*n* = 72) (Fig. 1). These geographic subregions were selected as the Hunter Valley; Brisbane and Clarence Rivers have been identified as biogeographic barriers to gene flow in koalas (Johnson et al. 2018). Figures were created using ggplot2 (v3.4.2) (Wickham 2016) in R 4.1.3 (Team 2022).

## Results

### Nucleotide diversity and selection analysis

We investigated diversity in 38 AMP genes (7 cathelicidins, 31 defensins) across 418 individuals. Six hundred and nine SNPs were retained following filtering. From these 59 SNPs were exonic, out of which 39 (66%) were non-synonymous (Table 1). For cathelicidins, all seven genes contained exonic SNPs of which 8 were synonymous and 13 were non-synonymous. Most of the non-synonymous SNPs (12 out of 13) were in the prepro region rather than the active mature peptide.

**Table 1:** Summary of nucleotide and allelic diversity. For Tajima’s *D* values, an asterisk indicates a p-value < 0.05. Phci is the precursor indicating the species, in this case, koala. CATH is cathelicidin; DEF is the defensins with DEFA indicating the alpha defensins and DEFB indicating the beta defensins. Splice refers to a SNP that is within 2 bp from an exon/intron boundary

Name of gene	Total SNPs	Intronic SNPs	Exonic SNPs	Non-synonymous exonic SNPs	Non-synonymous SNPs in active peptide	Stop gain	Splice	Number of alleles	Sites under negative selection	CNV?	Tajima’s *D*
PhciCATH1	24	23	1	1	0	0	0	2	0	N/A	− 0.50013
PhciCATH2	22	18	4	3	0	0	0	5	0	N/A	1.39557
PhciCATH3	24	21	3	2	1	0	0	3	0	N/A	1.02439
PhciCATH5	29	27	2	0	0	0	0	4	2	Duplication	1.93808
PhciCATH6	26	23	3	2	0	0	0	4	0	N/A	0.03613
PhciCATH7	27	25	2	2	0	0	0	3	0	N/A	− 0.81107
PhciCATH8	68	62	6	3	0	0	1	9	3	N/A	0.17117
PhciDEFA1	3	0	3	2	2	1	0	4	0	N/A	1.92027
PhciDEFA2	20	19	1	0	0	0	0	1	0	N/A	− 0.84986
PhciDEFB1	69	69	0	0	0	0	0	1	0	N/A	N/A
PhciDEFB10	124	123	1	1	1	0	0	2	0	N/A	1.39771
PhciDEFB11	2	0	2	2	2	0	0	3	0	N/A	0.67435
PhciDEFB12	5	0	5	4	4	1	0	5	0	N/A	2.66593*
PhciDEFB13	0	0	0	0	0	0	0	1	0	N/A	N/A
PhciDEFB14	40	39	1	0	0	0	0	2	0	N/A	1.12422
PhciDEFB15	0	0	0	0	0	0	0	1	0	N/A	N/A
PhciDEFB16	21	20	1	1	1	0	0	2	0	N/A	− 0.65634
PhciDEFB17	0	0	0	0	0	0	0	1	0	N/A	N/A
PhciDEFB18	1	0	1	1	0	0	0	2	0	N/A	− 0.79334
PhciDEFB19	2	0	2	2	2	0	0	4	0	N/A	0.49335
PhciDEFB20	3	0	3	2	2	0	0	3	1	N/A	0.325
PhciDEFB21	0	0	0	0	0	0	0	1	0	N/A	N/A
PhciDEFB22	0	0	0	0	0	0	0	1	0	N/A	N/A
PhciDEFB23	0	0	0	0	0	0	0	1	0	Deletion	N/A
PhciDEFB24	1	0	1	1	1	0	0	2	0	Duplication	− 0.39911
PhciDEFB25	0	0	0	0	0	0	0	1	0	N/A	N/A
PhciDEFB26	3	0	3	0	0	0	0	4	0	N/A	0.59922
PhciDEFB27	1	0	1	1	1	0	0	2	0	N/A	− 0.232
PhciDEFB28	1	0	1	1	1	0	0	2	0	N/A	2.06845
PhciDEFB29	0	0	0	0	0	0	0	1	0	N/A	N/A
PhciDEFB3	26	24	2	1	1	0	0	3	0	N/A	0.39386
PhciDEFB30	0	0	0	0	0	0	0	1	0	N/A	N/A
PhciDEFB4	3	0	3	2	0	1	0	4	0	N/A	0.75082
PhciDEFB5	58	57	1	1	0	0	0	2	0	N/A	− 0.63003
PhciDEFB6	0	0	0	0	0	0	0	1	0	N/A	N/A
PhciDEFB7	2	0	2	1	1	0	0	3	0	N/A	− 0.81084
PhciDEFB8	1	0	1	0	0	0	0	2	0	N/A	− 0.76548
PhciDEFB9	3	0	3	3	3	0	0	4	0	N/A	1.8104

For defensins, 8 synonymous and 26 non-synonymous exonic SNPs were identified amongst the 31 genes studied. Non-synonymous SNPs in the active peptide were more common in defensins compared to cathelicidins, with 13 out 31 genes containing at least one non-synonymous SNP (Table 1). This included deleterious mutations such as stop gains in alpha defensin PhciDEFA1 and beta defensins PhciDEFB12 and PhciDEFB4. In particular, the stop gain in PhciDEFA1 removes some of the conserved cysteines involved in the disulphide bonds responsible for peptide structure. Similarly, PhciDEFB16 has a SNP that affected one of the conserved cysteine residues. As these cysteine residues are responsible for forming the three disulphide bonds that characterise defensins, this would likely lead changes in structure and function.

Two out of seven cathelicidins had between two and three sites, and one defensin had a single site under negative selection (Table 1). Tajima’s D was only significant for one AMP (PhciDEFB12), suggesting that this defensin may be under balancing selection (Table 1).

### Allelic diversity and spatial structuring

The AMPs investigated here in this study contained between 1 and 9 alleles, with a mean of 2.6 alleles per AMP (Table 1). Eleven defensins, over a quarter of all peptides investigated, were invariant in all populations.

Allelic diversity decreased along a geographic gradient. Of the 27 peptides that did consist of multiple alleles, two were monomorphic in North QLD, four were monomorphic in South QLD, six were monomorphic in North NSW, six were monomorphic in Mid NSW, eight were monomorphic in South NSW, and fourteen in Victoria (Supplementary File 5). Most individuals were homozygous for most of the genes (Supplementary File 6).

**Fig. 2:**
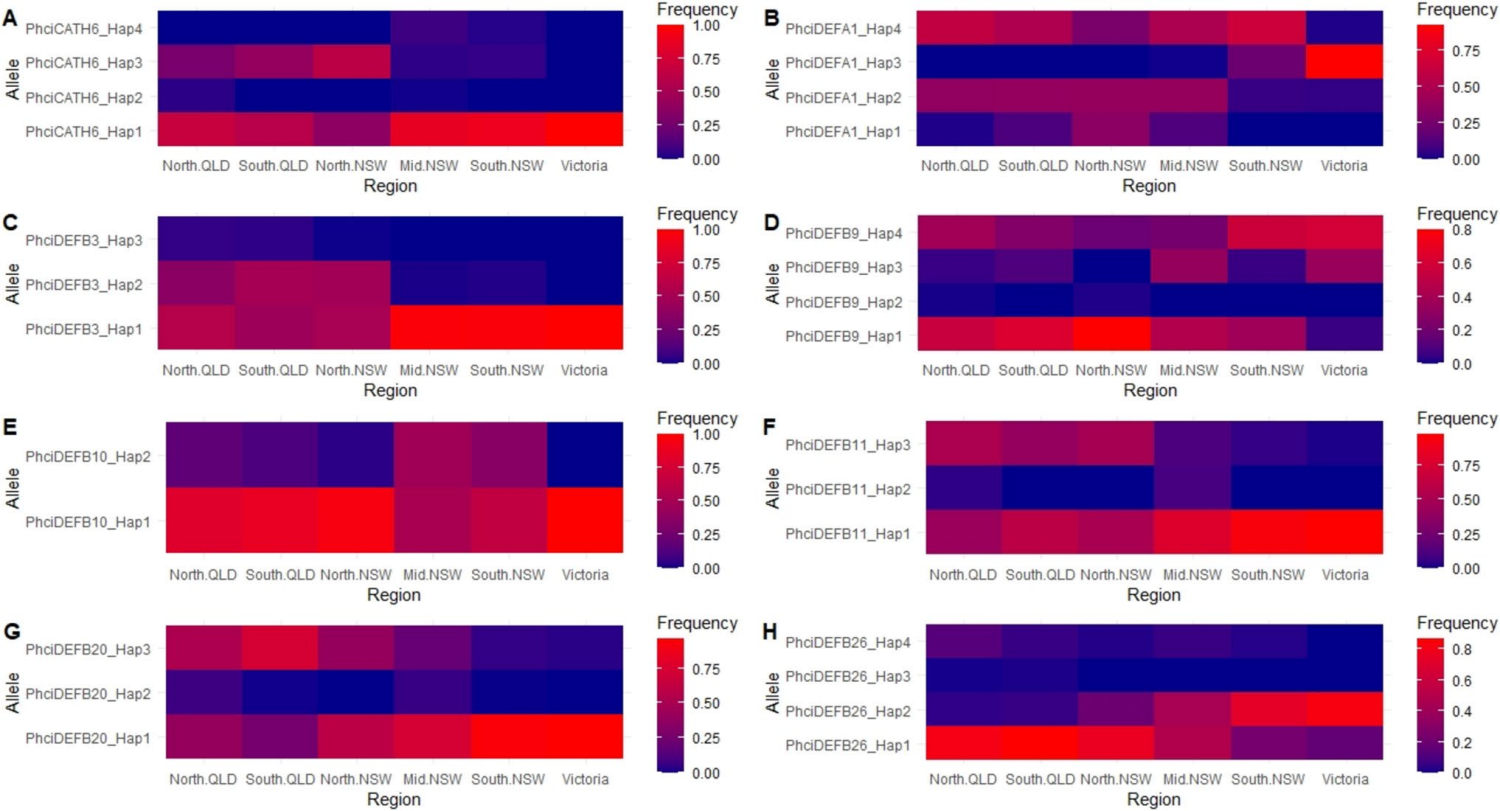
Heatmaps of allele frequencies in different geographic regions of **A** PhciCATH6, **B** PhciDEFA1, **C** PhciDEFB3, **D** PhciDEFB9, **E** PhciDEFB10, **F** PhciDEFB11, **G** PhciDEFB20, and **H** PhciDEFB26

Overall, allelic diversity was low: eleven peptides consisted of only two alleles, with a single predominant allele having a frequency > 0.70 in all regions (Supplementary File 5). However, there were some exceptions to this. For example, although PhciDEFB10_Hap1 was the major allele in all geographic regions, it had a much lower frequency in Mid NSW (0.526) than other populations (Fig. 2E).

For PhciCATH6, PhciDEFA1, PhciDEFB9, and PhciDEFB26, the major allele differed between geographic regions. PhciCATH6_Hap1 was predominant in all regions except for North NSW (Fig. 2A). PhciDEFA1_Hap4 was predominant in QLD and South NSW, PhciDEFA1_Hap3 was predominant in Victoria, while North and Mid NSW contained a balance of two or three alleles (Fig. 2B). PhciDEFB9_Hap1 was the most common in northern regions (QLD, North and Mid NSW), and PhciDEFB9_Hap4 was the most common allele in South NSW and Victoria (Fig. 2D). PhciDEFB26_Hap1 was most common in northern regions (QLD, North NSW), PhciDEFB26_Hap2 was most common in southern regions (South NSW and Victoria), while Mid NSW contained similar frequencies of both (0.491 of PhciDEFB26_Hap1 and 0.447 of PhciDEFB26_Hap2) (Fig. 2H).

### *In silico* predictions

14 AMPs contained non-synonymous SNPs in the active mature peptide region that were predicted to influence function (Table 2). This included eight peptides that had an increase in charge of one or greater and six peptides with a change in predicted antimicrobial or immunomodulatory activity.

**Table 2:**
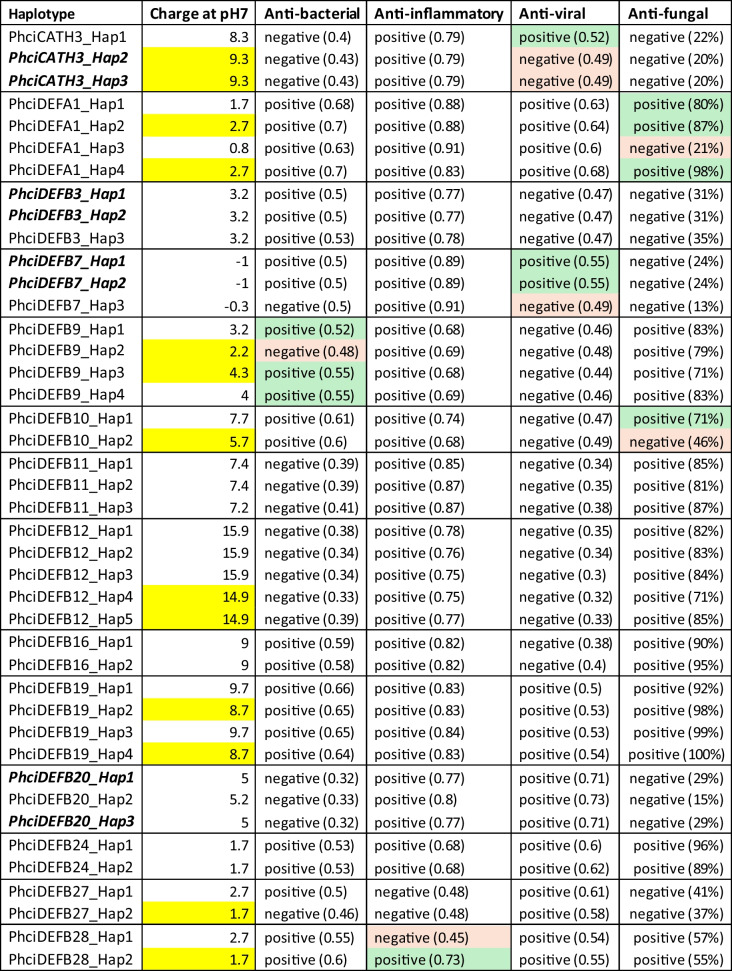
*In silico* predictions of the effect of non-synonymous SNPs on AMP function, with predicted changes in function highlighted in red (negative) or green (positive). Changes in charge greater than 1 are highlighted in yellow. Italics indicate that the two haplotypes have the same amino acid sequence in the active peptide region. Haplotype frequency was calculated using PHASE (version 2.1.1), anti-bacterial, anti-inflammatory, and anti-viral activity predicted using CSM-peptides webserver (Rodrigues et al. 2022) and anti-fungal activity predicted using the AntiFungal webserver (Zhang et al. 2021). The numbers and percentages in brackets indicate the probability of activity as either a frequency or a percentage depending on the webserver used. ‘Positive’ indicates a probability value > 0.50

Only one cathelicidin (PhciCATH3) contained non-synonymous SNPs in the active mature peptide (Table 2). PhciCATH3_Hap2 and PhciCATH3_Hap3 both had a G > R substitution which increased the charge at pH7 from + 8.3 to + 9.3. While PhciCATH3_Hap1 was predicted to have antiviral activity (Table 2), the other two alleles are not. PhciCATH3_Hap1 is the predominant allele present across the entire geographic range and the only allele present in Victoria. PhciCATH3_Hap3 showed the highest frequency in QLD, where it had a frequency of 0.234 in North QLD and 0.157 in South QLD.

Although koala defensins have not yet been tested *in vitro*, many are predicted to have antifungal activity (PhciDEFA1, PhciDEFB9, PhciDEFB10, PhciDEFB11, PhciDEFB12, PhciDEFB16, PhciDEFB19, PhciDEFB24, PhciDEFB28) (Table 2). Some defensins contained variants that altered this predicted activity. For example, PhciDEFB10_Hap2 had a K > E substitution that resulted in a lower charge at pH 7 (+ 5.7) than PhciDEFB10_Hap1 (+ 7.7). While PhciDEFB10_Hap1 was predicted to have antifungal activity, PhciDEFB10_Hap2 was not (Table 2). PhciDEFB10_Hap1 is also the predominant allele across the entire range (Fig. 2E) and the only allele present in Victoria. PhciDEFB10_Hap2 has a higher frequency in Mid NSW (0.474) and South NSW (0.353).

PhciDEFA1_Hap3 contains a stop codon resulting in the loss of two of the conserved cysteine residues. Interestingly, although this allele was not predicted to be anti-fungal like the other three alleles, it still had predicted anti-bacterial, anti-inflammatory, and anti-viral activity (Table 2). PhciDEFA1_Hap3 is rare across most of the range, but it is the major allele in Victoria (Fig. 2B).

Except for PhciDEFB27, all the AMPs were predicted to have anti-inflammatory properties but not all alleles (Table 2). For example, although PhciDEFB28_Hap2 is predicted to have anti-inflammatory properties but not PhciDEFB28_Hap1. PhciDEFB28_Hap2 is also the predominant allele, occurring at frequencies between 0.606 and 0.667 in every geographic region.

Of the four PhciDEFB9 alleles, all were predicted to have antifungal activity, and all but one (PhciDEFB9_Hap2) were predicted to have antibacterial activity. PhciDEFB9_Hap2 was extremely rare and only occurred at very low frequencies in North Queensland (0.011) and North NSW (0.020).

### CNVs

**Fig. 3:**
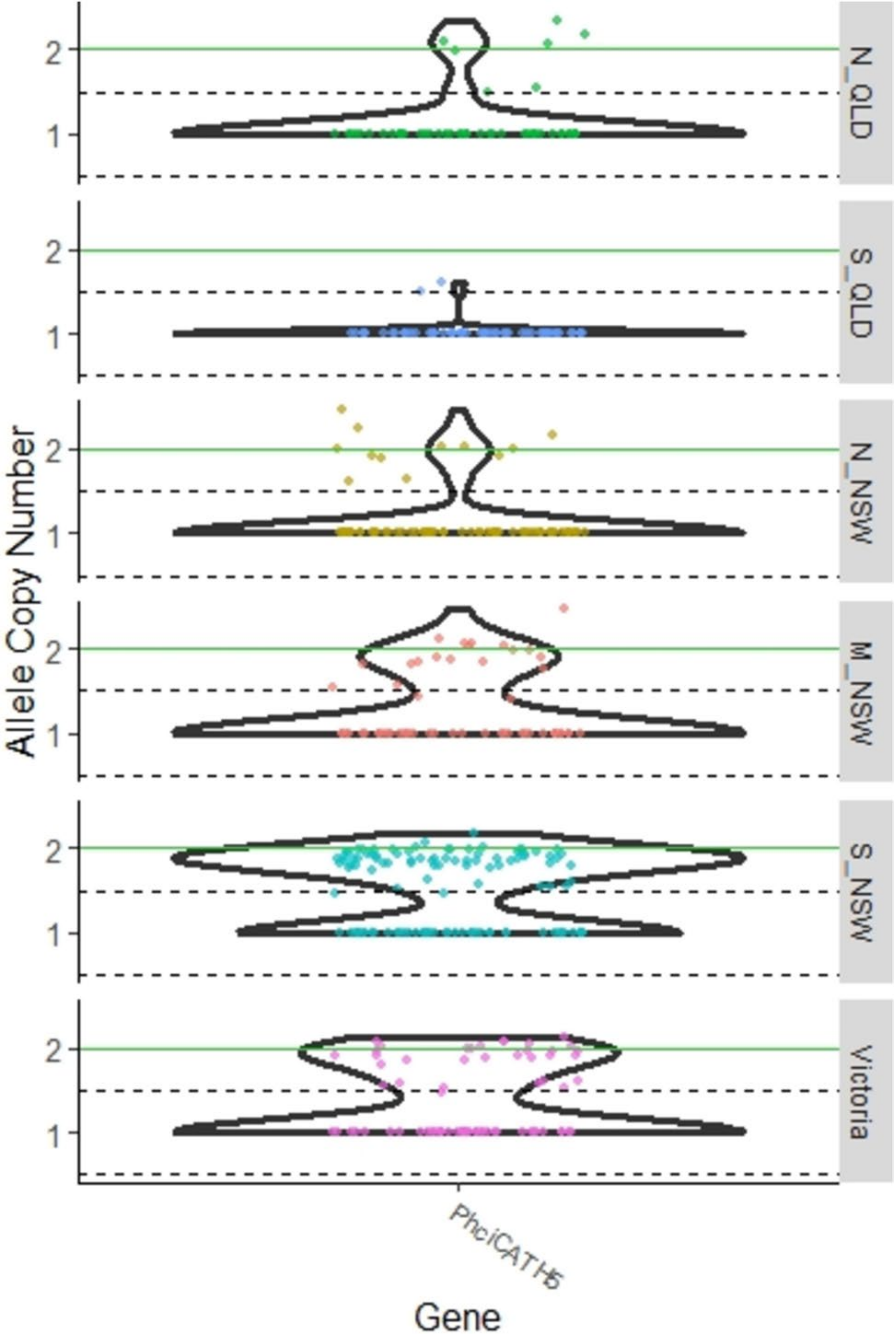
Violin plot depicting the most common CNV in koala AMPs (PhciCATH5) based on CNVnator analysis. Each dot represents an individual. Any individuals that did not have a statistically significant different normalised read depth are represented as having a normalised read depth equal to 1. Dotted lines represent the boundaries of a hemizygous deletion or duplication, and the green line represents the boundary of a whole gene duplication

In total, 177 copy number variants (CNVs) were retained following filtering (152 duplications and 25 deletions) (Supplementary File 4). The most common CNV involved a duplication of  PhciCATH5. Although this was found across the entire geographic range, it was most common in southern areas (Fig. 3). In QLD, it only occurred in 9 out of 101 individuals (9%), and in Northern NSW, it only occurred in 12 out of 76 (15%). However, in Mid NSW, it was found in 20 out of 57 (35%); in South NSW, it was found in 71 out of 112 (63%); and in Victoria, it was found in 32 out of 72 (44%). The normalised read depth of this region ranged from 1.4 to 2.5, with an average of 1.87 (Fig. 3). A normalised read depth of one represents the reference genome (i.e. a single copy of the gene, or two copies in a diploid organism) so these values suggest that whole gene duplications are common.

One defensin (PhciDEFB24) also exhibited duplications but it was much rarer. The duplication occurred in six individuals from Mid NSW, two from South QLD, and one from North QLD (Supplementary File 4). In these individuals, normalised read depth ranged from 1.6 to 2.7 also indicating a whole gene duplication.

PhciDEFB23 exhibited deletions in 25 individuals from across the entire range: five from North QLD, two from South QLD, 12 from North NSW, two from Mid NSW, three from South NSW, and one from Victoria. They had a normalised read depth around 0.5 (half the coverage of the reference genome) suggesting a hemizygous deletion (Supplementary File 4).

## Discussion

Here, we characterized AMP diversity across most of the koala range using 418 whole genomes from the east coast of Australia. We found non-synonymous SNPs in 14 AMPs that are predicted to change the function of the active mature peptide. We also found copy number variants involving duplications in two AMPs and deletions in one. Although AMP diversity has previously been investigated in birds and agricultural species, previous studies have been limited to studying a small number of genes and most of these have only looked at one or two populations. This represents the first comprehensive exploration of intraspecific AMP diversity in a wild mammal species across its range.

Overall, AMP allelic diversity was lower than in other immune families we have investigated such as MHC (Silver et al. 2024) and toll-like receptors (TLR) (Cui et al. 2025). This is not surprising given AMPs are not involved in antigen recognition but rather utilise physiochemical properties such as cationic charge to exert their antimicrobial effects. It is also consistent with previous studies that have indicated ancient genes of the innate immune system are more constrained than genes of the adaptive immune system (Chapman et al. 2016; Clark & Wang 1997).

The higher rate of SNPs observed in the defensins mirrors the findings of other studies (Supplementary File 1). Our findings that most non-synonymous SNPs in cathelicidins are in the prepro region rather than the active sequence are also consistent with the results of similar studies on pig and cattle cathelicidins (Ahn et al. 2022; Gillenwaters et al. 2009). This supports the idea that there are more constraints on the mature peptide than the prepro region.

Interestingly, no non-synonymous SNPs were identified in PhciCATH5, the only cathelicidin with documented antimicrobial activity *in vitro* (Peel et al. 2021). This may indicate strong selective pressure to conserve this function. Selection analysis provided further evidence of the constraints on cathelicidin evolution, with mostly negative selection shown (Table 1). This is similar to other studies that have shown negative selection to be more common than positive selection in cathelicidins in birds (Cheng et al. 2015). However, we note that due to the short length of AMP sequences, tests of selection have limited statistical power.

Some AMPs did, however, exhibit higher levels of polymorphism. This mirrors the results of a similar study in mallards, which showed that one allele is predominant for most defensins, but some have higher diversity (Chapman et al. 2016). Chapman et al. investigated five defensin genes in mallards and other species of Anatidae (ducks, geese, and swans). Out of these five genes, the study only found one (*AvBD3b*) with allele frequencies that differed between geographic regions. In contrast, we investigated 38 genes and found allelic frequency differences in seven defensins (PhciDEFA1, PhciDEFB3, PhciDEFB9, PhciDEFB11, PhciDEFB12, PhciDEFB20, and PhciDEFB26) and one cathelicidin (PhciCATH6) (Supplementary File 5). The high Tajima’s *D* value for PhciDEFB12 suggests this gene may be under balancing selection. Balancing selection is well-documented in MHC genes but not ubiquitous in other immune genes (Minias & Vinkler 2022). For example, while some TLR and AMP genes show signs of balancing selection, many others do not (Chapman et al. 2016, 2019; Cui et al. 2025; Hollox & Armour 2008; Kloch et al. 2018; Podlaszczuk et al. 2020). Our study provides evidence that although balancing selection is rare in koala AMPs, it does occur.

Overall, northern populations contained more alleles than southern population which is consistent with previous studies that have shown southern koalas have lower genetic diversity (Johnson et al. 2018; Lott et al. 2022). This is the result of population crashes caused by the fur trade in the late nineteenth century, leading to a genetic bottleneck (Lee et al. 2011). Northern and southern koala populations also display phenotypic variation across the range, including morphological (body size, fur depth) (Briscoe et al. 2015) and disease susceptibility (Legione et al. 2016; Patterson et al. 2015; Polkinghorne et al. 2013). Interestingly, despite their low genetic diversity, southern populations also tend to have reduced disease severity. This includes a lower prevalence of koala retrovirus (KoRV) (Simmons et al. 2012) and reduced severity of chlamydiosis (Legione et al. 2016; Patterson et al. 2015; Polkinghorne et al. 2013).

We recommend future studies investigate the functional effects of the different alleles identified here through population studies using disease metadata. Some of the AMPs that may have functional impacts include PhciDEFA1, PhciDEFB9, PhciDEFB10, and PhciCATH6. PhciDEFA1_Hap3 allele has a stop codon which is predicted to have a deleterious function. Interestingly, although our *in silico* results predict that this allele has lost its antifungal activity, it was the most common allele in Victoria. As PhciDEFA1 is expressed in the lactating mammary gland and likely present in the milk, we hypothesise that it may be involved in protecting the young (Peel et al. 2024).

PhciDEFB9_Hap1 is the most common allele in northern regions (QLD, North and Mid-NSW), while PhciDEFB9_Hap4 is most common in southern regions (South NSW and Victoria). Although our *in silico* testing predicted the different alleles have both anti-bacterial and anti-fungal activity, it is possible they vary in specificity and potency. We recommend future studies test these alleles in vitro.

PhciDEFB16_Hap2 has a SNP that changes one of the conserved cysteines that may lead to change in structure and consequently function. This haplotype was rare and only found in northern regions (frequency of 0.011 in North QLD and 0.083 in South QLD) (Supplementary File 5). The properties of PhciDEFB16 have not yet been characterised although our in silico testing predict both alleles have antibacterial, antifungal, and anti-inflammatory properties (Table 2). It is an ortholog of the mouse defensin DEFB33, which is expressed in the testes and may play a role in reproduction (Patil et al. 2005). It is possible that PhciDEFB16 may play a similar role in koalas; however, further studies should investigate its gene expression in koala testes transcriptomes to confirm this.

Naturally occurring AMPs have long been deemed promising candidates for drug development (Lazzaro et al. 2020). In many cases, the AMP sequence serves as a template that can be modified for optimised activity such as increased activity or specificity. This is often achieved through minor amino acid substitutions (Higgs et al. 2007; Molhoek et al. 2010; Porto & Alencar 2023). For example, substituting lysine for arginine has increased the antiviral properties of the scorpion peptide BmKn2, as well as the amphibian peptides maximim H5 and dermaseptin S9 (Chen et al. 2012; Wang et al. 2010). While substitutions leading to an increase in net peptide charge sometimes improve antimicrobial potency and specificity (Higgs et al. 2007; Molhoek et al. 2010; Porto & Alencar 2023), an excessive increase can have the opposite effect by altering structure (Wang et al. 2019). By identifying different alleles of the various peptides in addition to the reference sequence, we have expanded the number of template sequences that can tested and optimised for drug development.

In addition to SNPs, CNVs such as duplications and deletions represent a major source of genetic variability between individuals. CNVs are often more common in immune genes than other regions of the genome (Bickhart et al. 2012; De Smith et al. 2009). The pleiotropic nature of AMPs means that such mutations can influence a wide variety of traits, including disease susceptibility, fertility, coat colour, and production traits (da Silva et al. 2016; Machado & Ottolini 2015; Yue et al. 2013; Zhang et al. 2009). Defensins in particular are recognised as being highly polymorphic in copy number, with duplications often involving whole clusters of genes. For example, the 8p23.1 locus in humans has been identified as a hotspot of genetic variation due to its high mutation rate (Bakar et al. 2009). This region encodes multiple defensins (DEFB4, DEFB103, DEFB104, DEFB105, DEFB106, DEFB107), with individual copy number ranging from two to twelve (Hollox et al. 2003). High copy numbers in this region have been associated with inflammatory diseases such as psoriasis and hidradenitis suppurativa (Giamarellos-Bourboulis et al. 2016; Hollox et al. 2008).

CNVs involving cathelicidins have also been reported in livestock (Bickhart et al. 2012; Jeon et al. 2019) although these are less common than defensins. One example is the cattle cathelicidin indolicidin, encoded by the CATHL4 gene. Indolicidin induces cell death in the parasite *Leishmania donovani*, the pathogen responsible for Leishmaniasis (Bera et al. 2003). This gene has reported to have undergone recent duplications in indicine cattle, who have a higher copy number than taurine cattle (Bickhart et al. 2012). Interestingly, indicine cattle also have a higher parasite resistance than taurine cattle (Berman 2011).

Similarly, CNVs were less common in koala cathelicidins than defensins. The only cathelicidin duplication we found was in PhciCATH5, a cathelicidin with activity against a range of microbes including *C. pecorum* (Peel et al. 2021). Chlamydia can manifest clinically as ocular and urogenital infections, resulting in blindness and infertility (Polkinghorne et al. 2013). Although *C. pecorum* affects koalas across their entire geographic range, disease prevalence and severity are both greater in northern populations (Legione et al. 2016; Patterson et al. 2015; Polkinghorne et al. 2013). Unlike koalas from NSW and QLD, *C. pecorum* has not been detected in ocular swabs from Victorian koalas (Patterson et al. 2015). Although mild ‘wet-bottom’ disease (urine staining the rump due to urinary tract infection) has been reported in Victorian koalas, ocular disease has not (Patterson et al. 2015). Interestingly, the PhciCATH5 duplication was more common in southern NSW and Victoria, where it was found in 63% and 44% of the sequenced genomes, respectively. PhciCATH5 duplications may be linked to the selective pressure of chlamydia, similar to the expansions observed in cattle indolicidin. However, additional work is required to confirm this pattern. We recommend population studies using disease metadata to determine if there is a significant association between PhciCATH5 duplications and disease susceptibility.

## Conclusion

In conclusion, we have characterised AMP diversity across a species range for the first time in mammalian wildlife and identified differences between populations at the nucleotide, amino acid, and copy number levels. We have also identified variants of interest for further investigation through population studies and *in vitro* testing.

## Supplementary Information

Below is the link to the electronic supplementary material.

Supplementary file1 (XLSX 20 KB)

Supplementary file2 (XLSX 32 KB)

Supplementary file3 (FA 27 KB)

Supplementary file4 (XLSX 37 KB)

Supplementary file5 (XLSX 21 KB)

Supplementary file6 (XLSX 18 KB)

Supplementary file7 (PDF 867 KB)

Supplementary file8 (PDF 734 KB)
