# Supplementary material for: AMPed up immunity: 418 whole genomes reveal intraspecific diversity of koala antimicrobial peptides
Source: Immunogenetics. 2025 Jan 8;77(1):11. doi: 10.1007/s00251-024-01368-2 (PMC11711154; doi:10.1007/s00251-024-01368-2)
Supplement: Supplementary file 7 — Supplementary file7 (PDF 867 KB) [file 251_2024_1368_MOESM7_ESM.pdf]

Supplementary File 7. 3D visualisations of peptides with non-synonymous SNPs in the active peptide regions. Figures are of the active peptide with amino acid changes highlighted

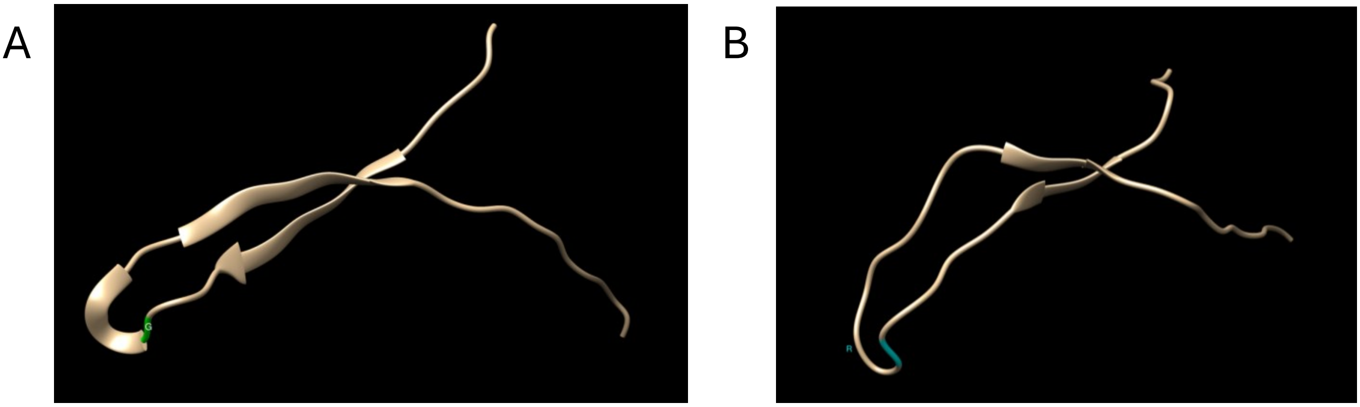

Figure 1. A. PhciCATH3_Hap1 B. PhciCATH3_Hap2

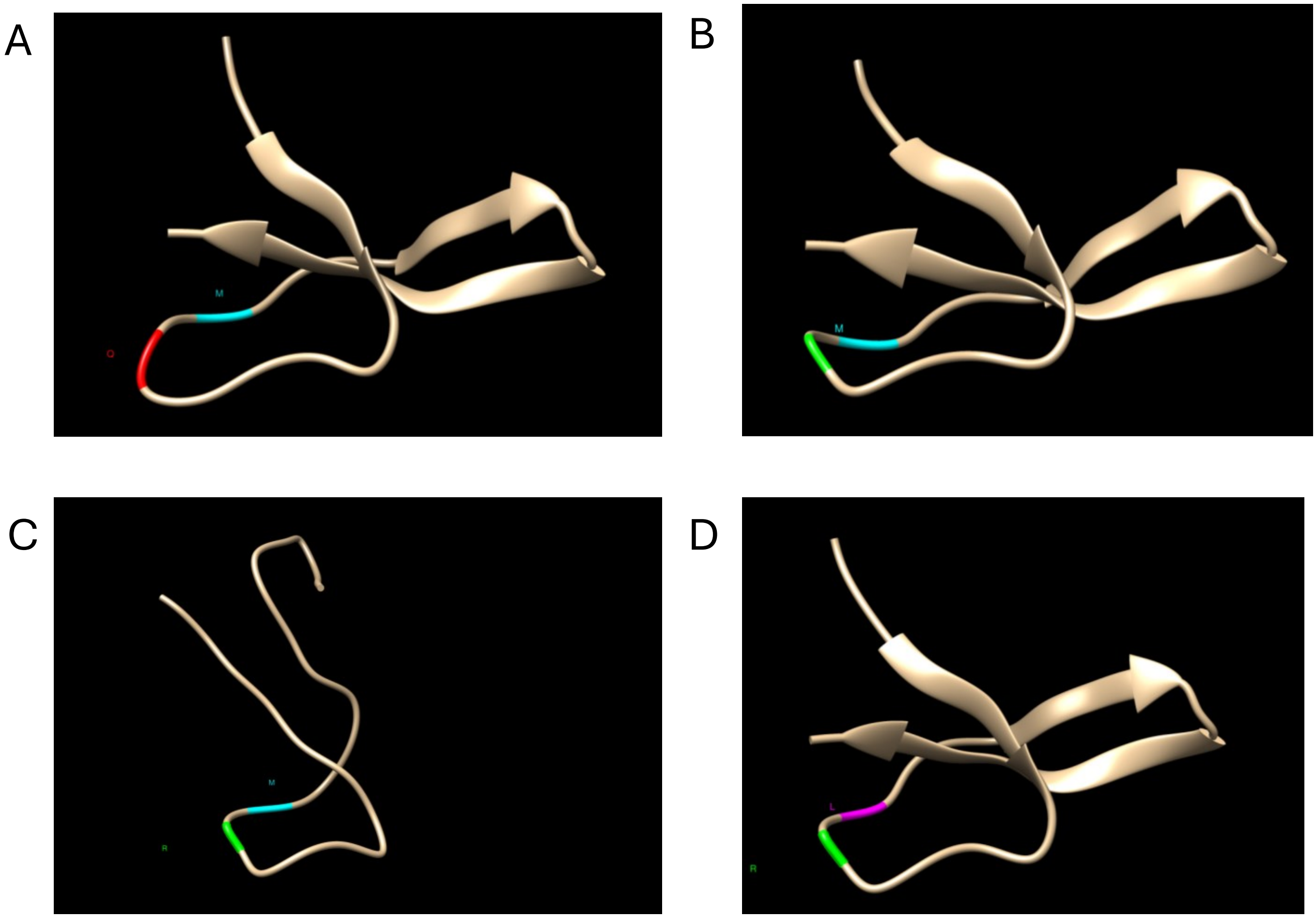

Figure 2. A. PhciDEFA_Hap1 B. PhciDEFA1_Hap2 C. PhciDEFA1_Hap3 D. PHciDEFA1_Hap4.

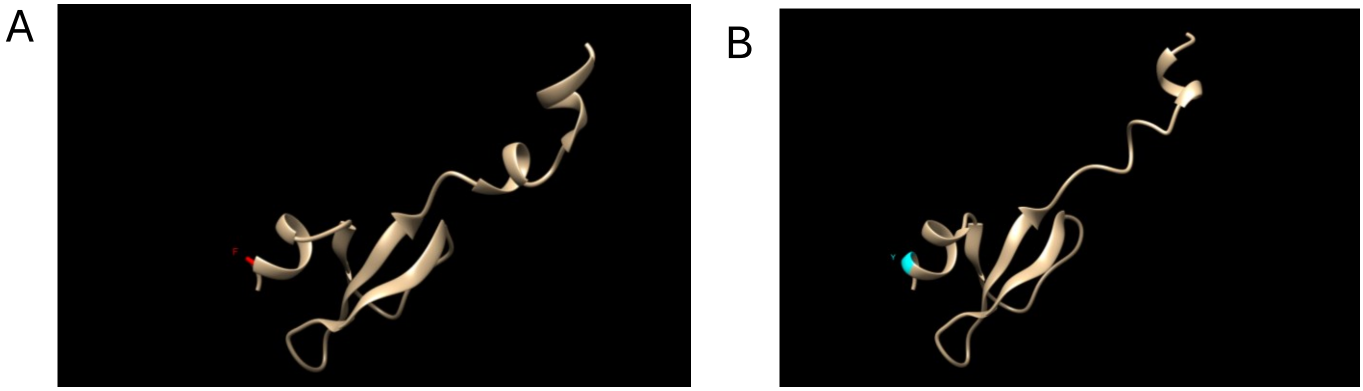

Figure 3. A. PhciDEFB3_Hap1. B. PhciDEFB3_Hap3

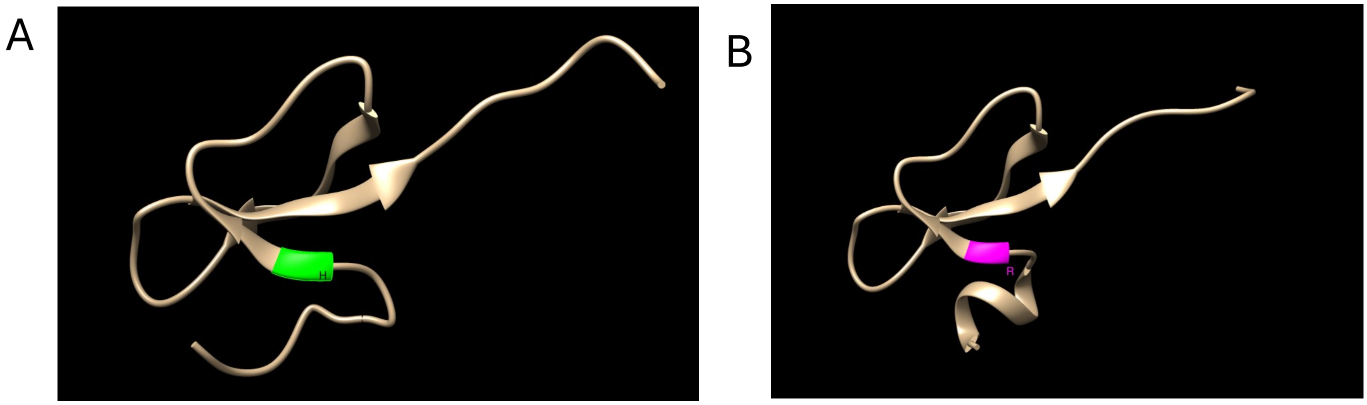

Figure 4. A. PhciDEFB7_Hap1 B. PhciDEFB7_Hap3

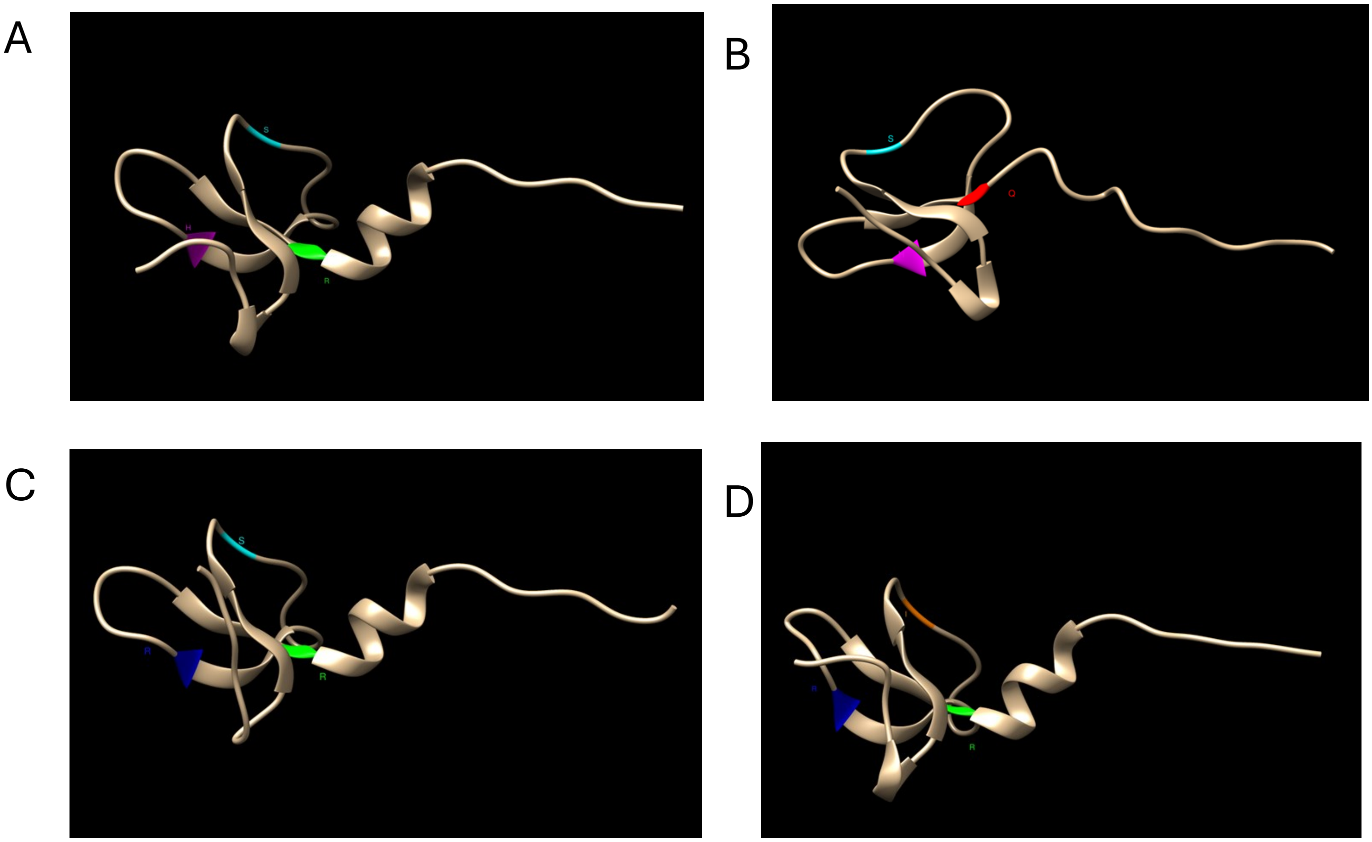

Figure 5. A. PhciDEFB9_Hap1 B. PhciDEFB9_Hap2 C. PhciDEFB9_Hap3 D. PhciDEFB9_Hap4

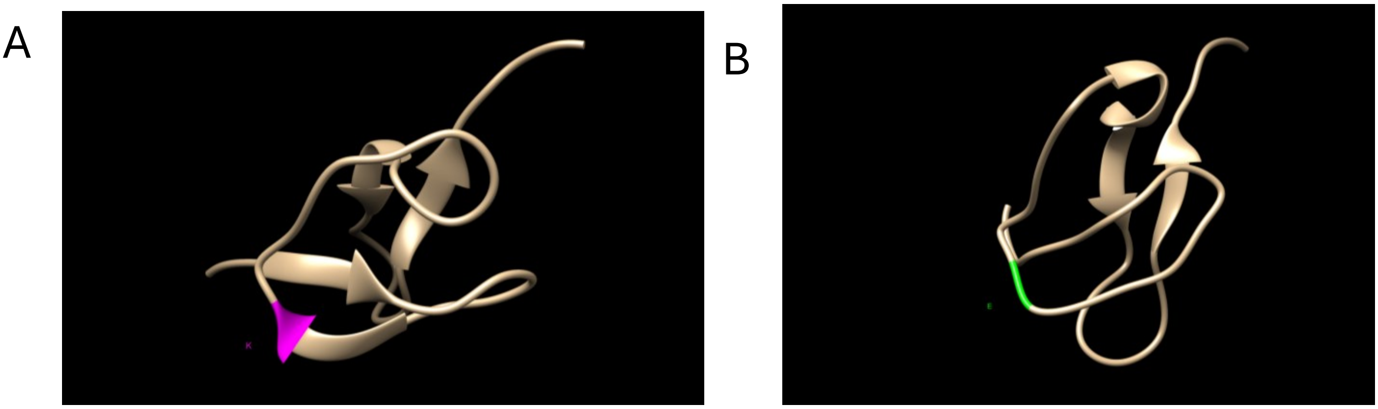

Figure 6. A. PhciDEFB10_Hap1 B. PhciDEFB10_Hap2

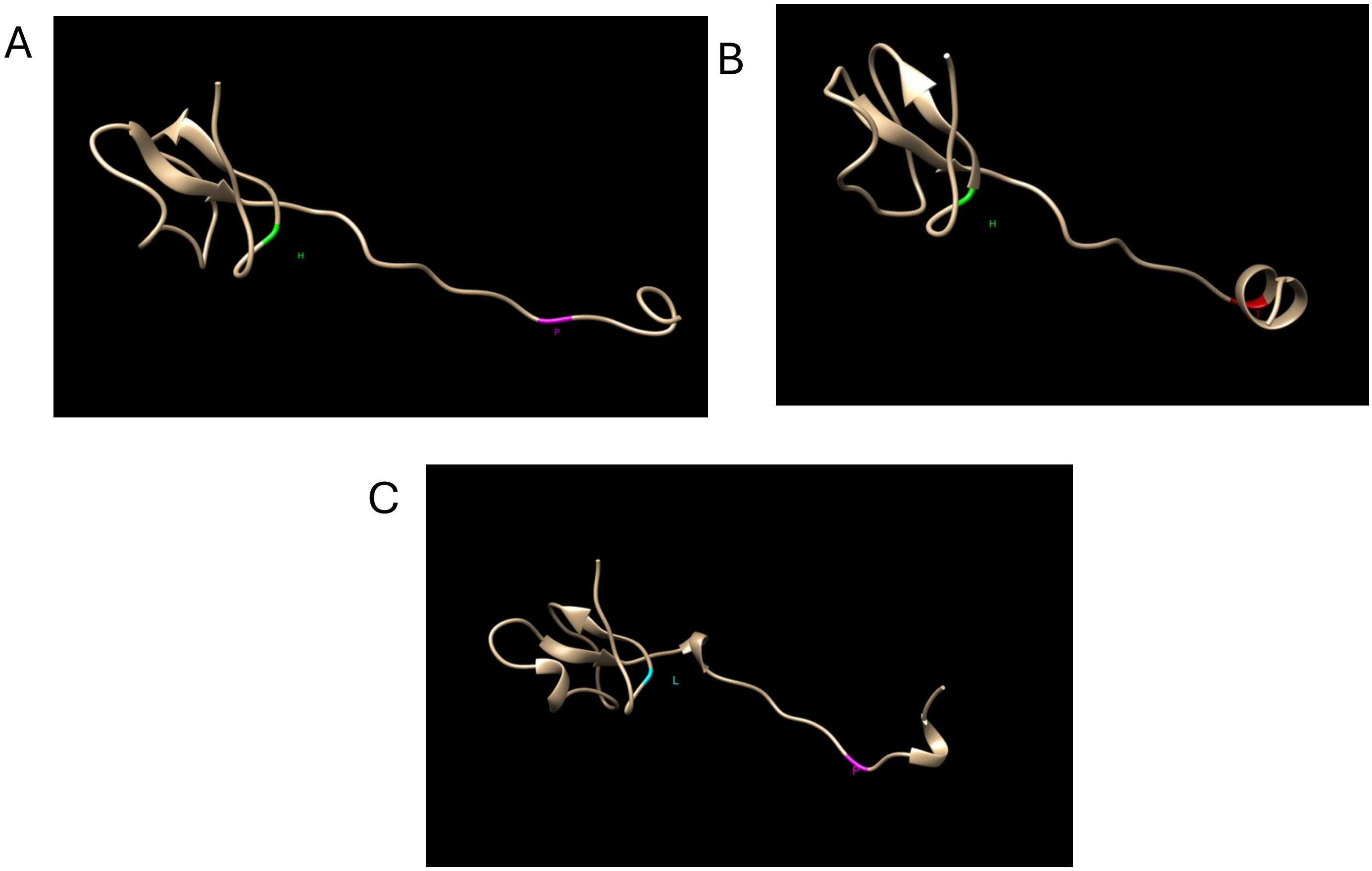

Figure 7. A. PhciDEFB11_Hap1 B. PhciDEFB11_Hap2 C. PhciDEFB11_Hap3

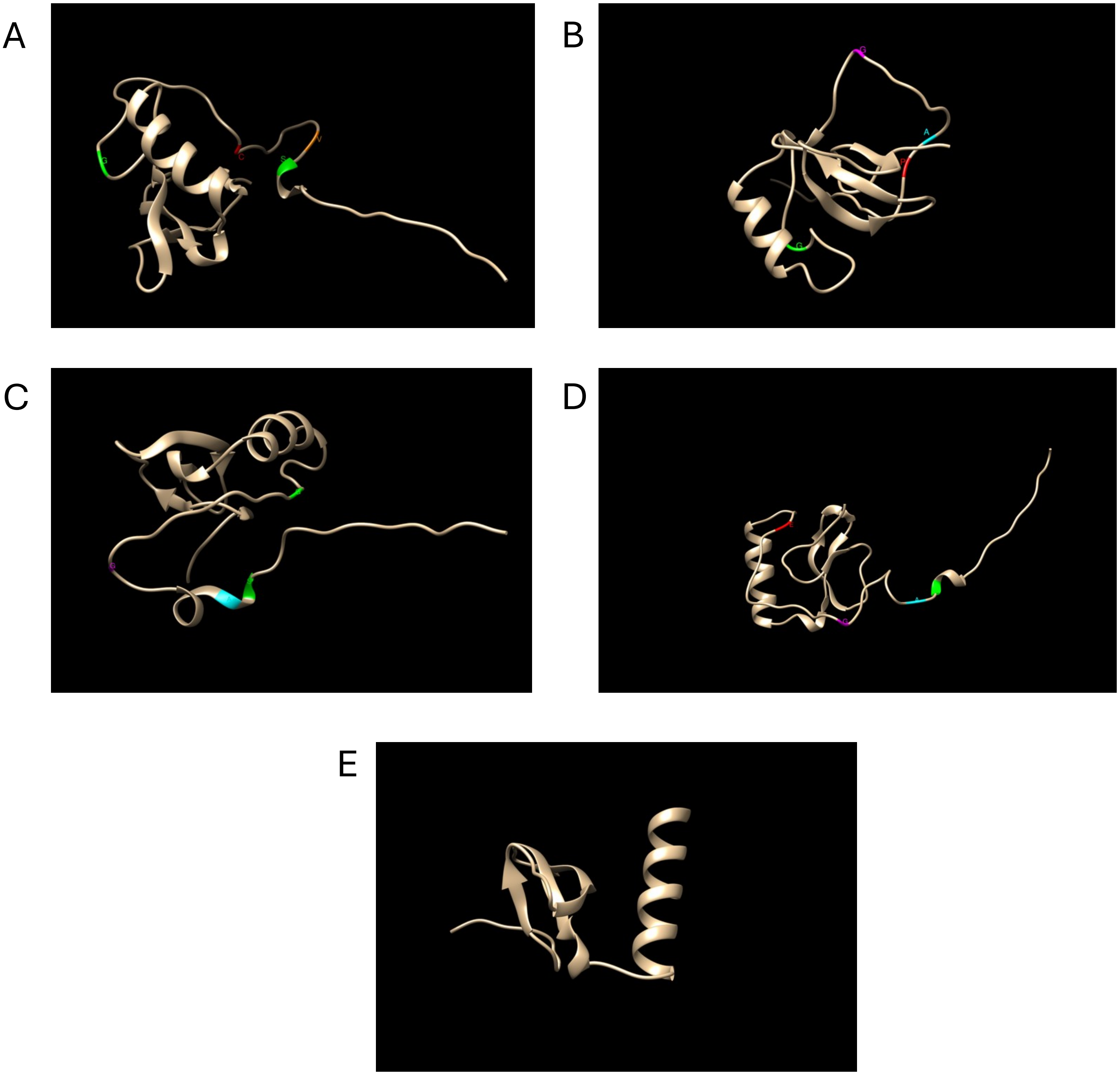

Figure 8. A. PhciDEFB12_Hap1 B. PhciDEFB12_Hap2 C. PhciDEFB12_Hap3 D. PhciDEFB12_Hap4 E. PhciDEFB12_Hap5

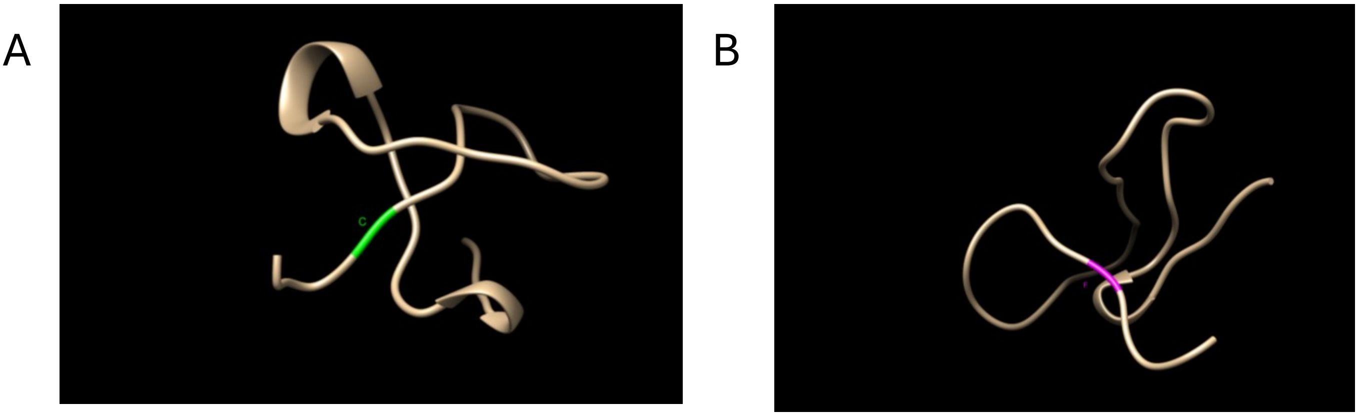

Figure 9. A. PhciDEFB16_Hap1 B. PhciDEFB16_Hap2

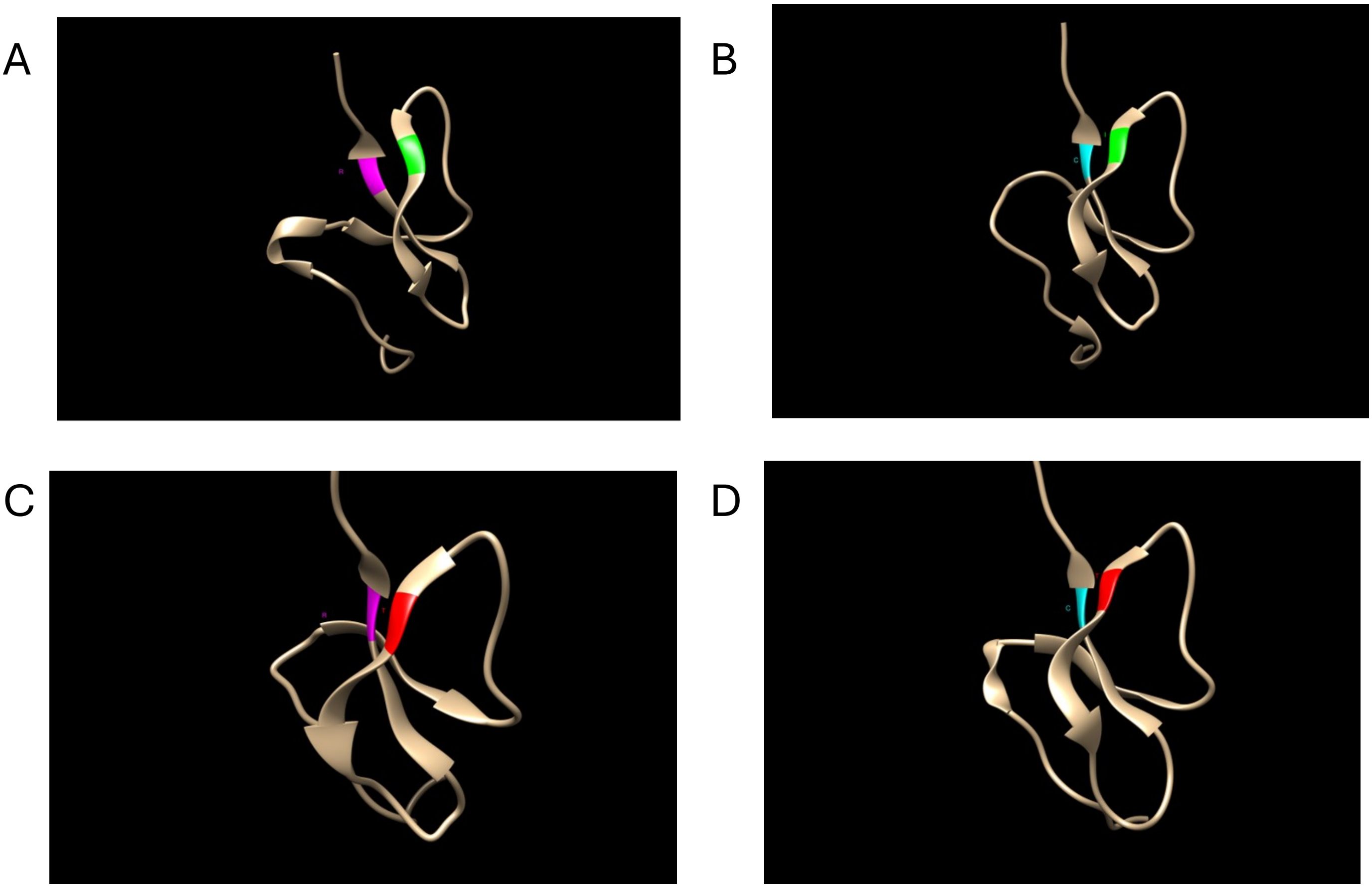

Figure 10. A PhciDEFB19_Hap1 B. PhciDEFB19_Hap2 C. PhciDEFB19_Hap3 D. PhciDEFB19_Hap4.

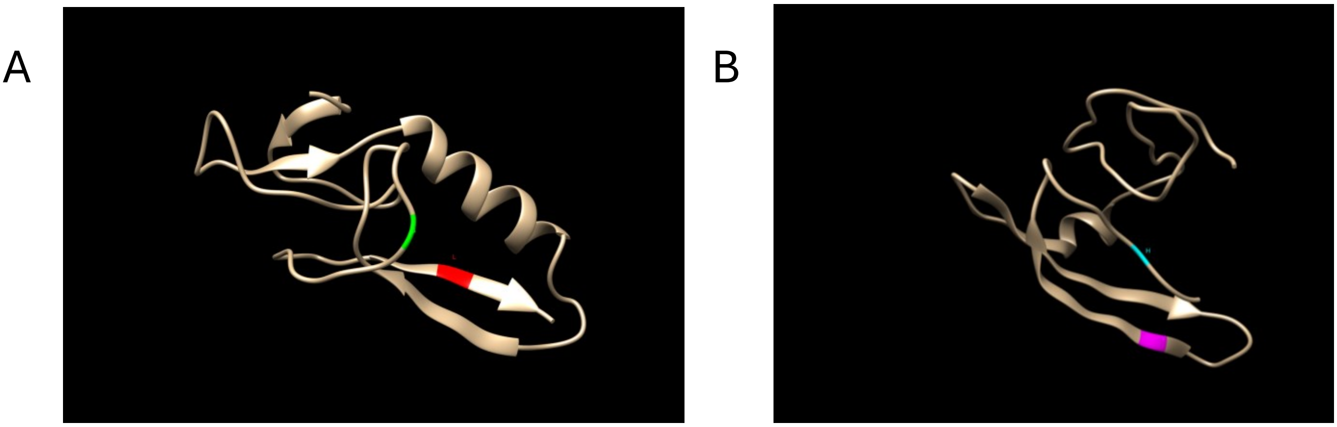

Figure 11. A PhciDEFB20_Hap1 B. PhciDEFB20_Hap2

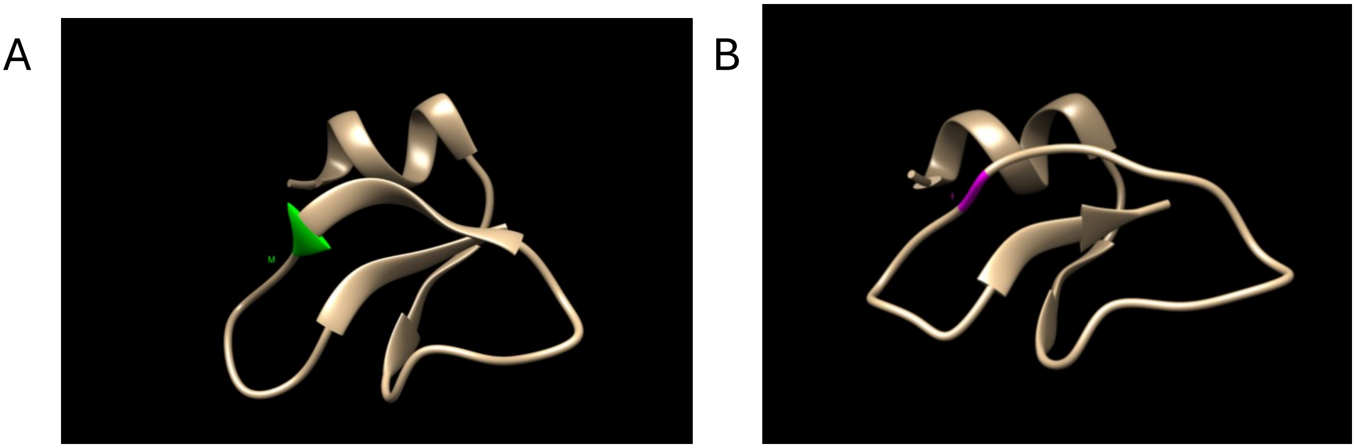

Figure 12. A PhciDEFB24_Hap1. B PhciDEFB24_Hap2

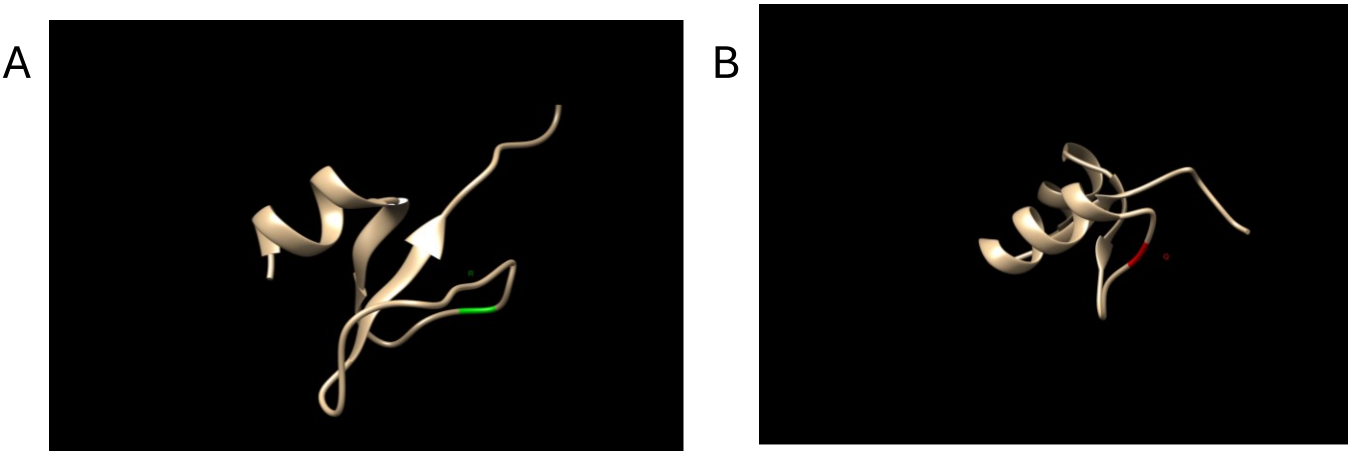

Figure 13. A. PhciDEFB27_Hap1 B. PhciDEFB27_Hap2.

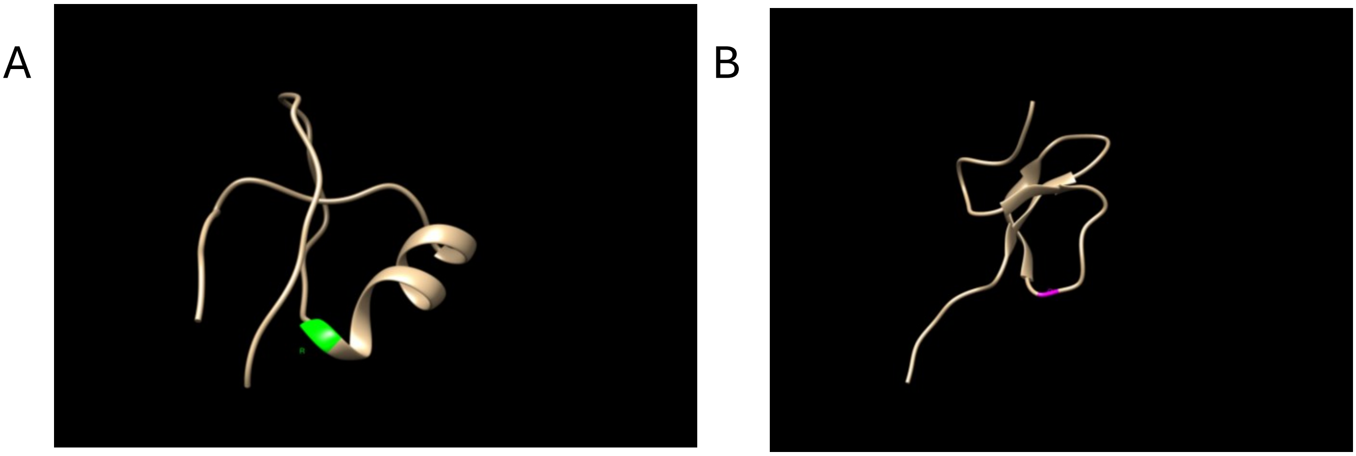

Figure 14. A PhciDEFB28_Hap1. B. PhciDEFB28_Hap2
