# Supplementary material for: AMPed up immunity: 418 whole genomes reveal intraspecific diversity of koala antimicrobial peptides
Source: Immunogenetics. 2025 Jan 8;77(1):11. doi: 10.1007/s00251-024-01368-2 (PMC11711154; doi:10.1007/s00251-024-01368-2)

Supplementary File 8. 3D visualization of (A) PhciCATH3_Hap1 and (B) PhciCATH3_Hap2 showing surface charge distribution changes with positively charged regions in blue and negatively charged regions in red.

A

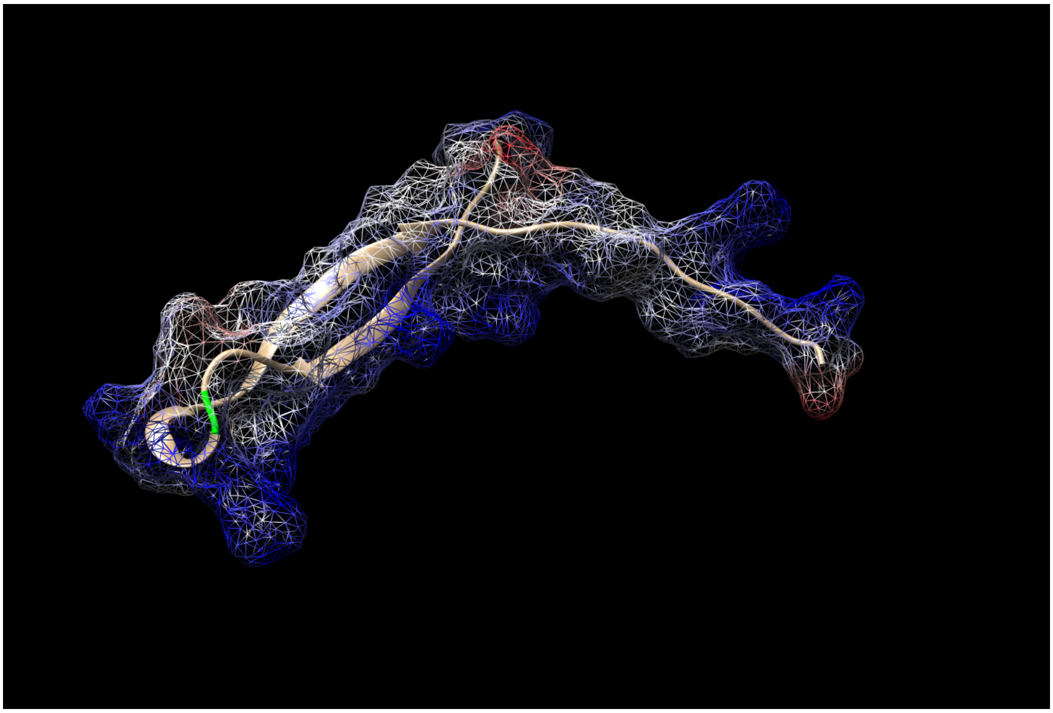

B

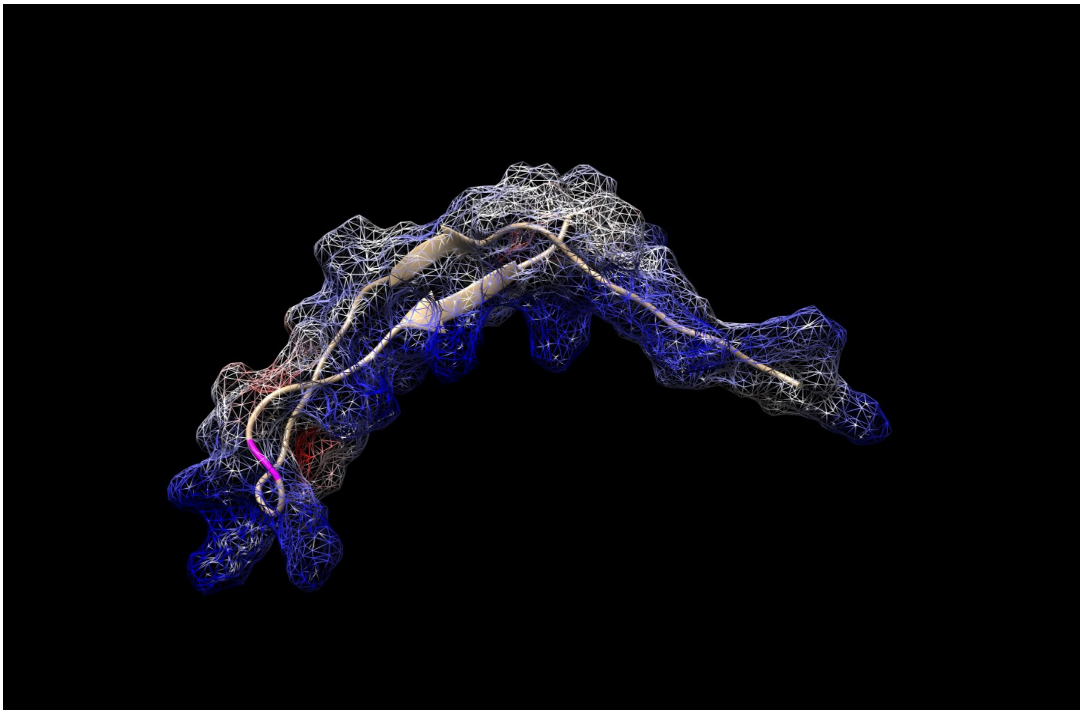

Supplement: Supplementary file 8 — Supplementary file8 (PDF 734 KB) [file 251_2024_1368_MOESM8_ESM.pdf]
